# An observational study on trajectories and outcomes of chronic low back pain patients referred from a spine surgery division for chiropractic treatment

**DOI:** 10.1186/s12998-018-0225-8

**Published:** 2019-02-05

**Authors:** Brigitte Wirth, Fabienne Riner, Cynthia Peterson, Barry Kim Humphreys, Mazda Farshad, Susanne Becker, Petra Schweinhardt

**Affiliations:** 1 Integrative Spinal Research Group, Department of Chiropractic Medicine, Balgrist University Hospital, Forchstr. 340, 8008 Zurich, Switzerland; 2 Spine Division, Department of Orthopedics, Balgrist University Hospital, Zurich, Switzerland; 3 Department of Cognitive and Clinical Neuroscience, Central Institute of Mental Health, Medical Faculty Mannheim, Heidelberg University, Mannheim, Germany

**Keywords:** Chiropractic, Low back pain, Outcome, Surgery

## Abstract

**Background:**

A close collaboration between surgeons and non-surgical spine experts is crucial for optimal care of low back pain (LBP) patients. The affiliation of a chiropractic teaching clinic to a university hospital with a large spine division in Zurich, Switzerland, enables such collaboration. The aim of this study was to describe the trajectories and outcomes of patients with chronic LBP referred from the spine surgery division to the chiropractic teaching clinic.

**Methods:**

The patients filled in an 11-point numeric rating scale (NRS) for pain intensity and the Bournemouth Questionnaire (BQ) (bio-psycho-social measure) at baseline and after 1 week, 1, 3, 6 and 12 months. Additionally, the Patient’s Global Impression of Change (PGIC) scale was recorded at all time points apart from baseline. The courses of NRS and BQ were analyzed using linear mixed model analysis and repeated measures ANOVA. The proportion of patients reporting clinically relevant overall improvement (PGIC) was calculated and the underlying factors were determined using logistic regression analyses.

**Results:**

Between June 2014 and October 2016, 67 participants (31 male, mean age = 46.8 ± 17.6 years) were recruited, of whom 46 had suffered from LBP for > 1 year, the rest for > 3 months, but < 1 year. At baseline, mean NRS was 5.43 (SD 2.37) and mean BQ was 39.80 (SD 15.16) points. NRS significantly decreased [F(5, 106.77) = 3.15, *p* = 0.011] to 4.05 (SD 2.88) after 12 months. A significant reduction was not observed before 6 months after treatment start (*p* = 0.04). BQ significantly diminished [F(5, 106.47) = 6.55, *p* < 0.001] to 29.00 (SD 17.96) after 12 months and showed a significant reduction within the first month (*p* < 0.01). The proportion of patients reporting overall improvement significantly increased from 23% after 1 week to 47% after 1 month (*p* = 0.004), when it stabilized [56% after 3 and 6 months, 44% after 12 months]. Reduction in bio-psycho-social impairment (BQ) was of higher importance for overall improvement than pain reduction.

**Conclusions:**

Chiropractic treatment is a valuable conservative treatment modality associated with clinically relevant improvement in approximately half of patients with chronic LBP. These findings provide an example of the importance of interdisciplinary collaboration in the treatment of chronic back pain patients.

## Background

Low back pain (LBP) is globally the leading cause for years lived with disability [1]. The point prevalence and 1-month prevalence are estimated to be around 12 and 23%, respectively [2]. The majority of cases have low levels of disability, but combined with the minority of highly disabled LBP-patients, LBP causes high societal and economic burden [3], comparable to cardio-vascular disease in high-income countries [4]. Less than 20% of the LBP patients seen by a surgeon typically need a surgical solution [5], but the challenge is the reliable identification of surgical indications [6]. Thus, close collaboration between spine surgeons and non-surgical spine experts, such as chiropractors, is crucial.

In some countries where chiropractic belongs to complementary and alternative medicine [7–9], some skepticism exists towards chiropractors, e.g. by orthopedic surgeons in North America [10] or general practitioners in Australia [11]. In Switzerland, good inter-professional collaboration between chiropractors and other medical specialists has already been reported in the Swiss job analysis survey in 2009 [12]. Additionally, chiropractic medicine is included as one of the five academic medical disciplines in Switzerland (medicine, dentistry, veterinary medicine, pharmacology, chiropractic medicine) and the chiropractic program is part of the Faculty of Medicine at the University of Zurich [12]. The chiropractic students complete a bachelor of medicine degree, in parallel to chiropractic specialty courses, before they enter the chiropractic master program. During the master program, the chiropractic students complete a 6 months professional internship in a teaching clinic that is affiliated to a mainly orthopedic university hospital with a large spine division, which offers the unique opportunity for spine surgeons to refer patients directly to the chiropractor, if conservative treatment is deemed to be appropriate.

The aim of this analysis was to study the trajectories and outcomes of patients with chronic low back pain (LBP) referred from the spine division to the chiropractic teaching clinic over the course of 12 months after start of chiropractic treatment.

## Methods

Patients referred from the spine surgery division to the chiropractic teaching clinic filled in a numeric rating scale (NRS) for present pain intensity and the Bournemouth Questionnaire (BQ), a bio-psycho-social outcome measure (maximum score = 70 points), at baseline (before the first chiropractic treatment) and after 1 week, 1, 3, 6 and 12 months. The BQ is a valid and reliable questionnaire that covers the multidimensionality of musculoskeletal pain in seven items [13]. Additionally, they completed the Patient’s Global Impression of Change (PGIC) scale at all time points apart from baseline. Patient rating of overall improvement as assessed by the PGIC is recommended as one of four core domains for chronic pain outcomes, besides pain intensity, and physical and emotional functioning [14]. The PGIC is a seven point Likert-scale with the extremes “much worse” and “much better” [15]. According to previous literature [16, 17], only the two highest categories (“much better” and “better”) were defined as clinically relevant improvement. After giving written informed consent, the questionnaires were administered to the patients by the treating chiropractor immediately before the first treatment. Patients then chose whether they preferred to answer the questionnaires at the follow-up time points via email or phone. If phone contact was preferred, a trained research assistant who did not know the patient conducted short telephone interviews at each time point, irrespective of whether the patient was still in chiropractic treatment or not. If online contact was chosen, survey invitations were sent to the participants at each time point using the software REDCap (version 8.3.2), a secure web-based application designed to support data capture for research studies [18]. This study was approved by the Ethics review board of the Canton of Zurich (EK-16/2009; update PB_2017–00402).

### Statistics

For describing the course of the NRS- and BQ-scores, missing values [in the follow-up assessments between *N* = 7/10.4% (NRS at 6 months) and *N* = 13/19.4% (PGIC at 12 months)] were handled in two ways to get a comprehensive picture [19]: (1) Linear mixed model analysis (LMM) (time as fixed factor) was performed and (2) repeated measures ANOVA with multiply imputed data (five imputations) was conducted. This analysis was run with each of the five imputations. Post hoc, Bonferroni correction was applied in both analyses.

Descriptive statistics were used to describe the proportion of patients who reported clinically relevant overall improvement in the PGIC at each time point and the McNemar test was used to test for significant differences between time points. In order to report “true” improvement, complete-case analysis was used for these analyses. With imputed values, a series of binary logistic regression analyses were conducted to investigate the importance of changes in NRS- and BQ-values for self-reported improvement (dichotomized PGIC data as dependent variable; 0 = not improved, 1 = improved, categories “much better” and “better” [16, 17]). To differentiate between the importance of pain reduction (NRS) and reduction in bio-psycho-social impairment (BQ), in a first step NRS values and BQ total scores were entered into the model (model 1). In a consecutive step, to investigate which component of the bio-psycho-social composite was most meaningful for overall change, the seven single BQ-items were entered into model 2, if BQ total score emerged as significant from model 1. Similarly, NRS values were entered into model 2 only if they were significant in model 1. For these analyses pooled data, resulting of 5 imputations, were used apart from Nagelkerke R^2^. All analyses were conducted in SPSS Statistics 21.0 (SPSS, Chicago, IL, USA) and the significance level α was set at 0.05.

## Results

Between June 2014 and October 2016, 67 patients with chronic (> 3 months) LBP (31 male, mean age = 46.8 ± 17.6 years) were referred from the spine surgeons to the chiropractic teaching clinic. The majority of the patients (*N* = 46) had been experiencing LBP for more than 1 year; the rest for more than 3, but less than 12 months. Twelve patients had previously undergone back surgery. The MRI of 31 patients showed degenerative changes, such as disc degeneration and stenosis. However, most MRI signs apart from Modic type 1 changes and intense, extensive zygapophyseal edematous changes are reported to be poorly correlated with LBP [20]. Similarly, the degree of stenosis is related to neurologic impairment, but unrelated to pain intensity and functional disability [21]. Thus, these patients were summarized here in a category ‘non-specified LBP’, together with 14 patients, whose MRI did not show any abnormal signs. Further pathologies were radiculopathy (*N* = 5), spondylolisthesis (*N* = 3), and osteoporosis (*N* = 2). Of these patients, 35% (*N* = 22, 4 missing values) took analgesic medication at baseline. For 52% (*N* = 34, 1 missing value) of the patients, the treating chiropractor judged their general health as good, for 41% (*N* = 27, 1 missing value) as average and for 8% (N = 5, 1 missing value) as poor. The median of the number of chiropractic consultations was 8 (interquartile range = 6.0) with 3 patients treated more than 20 times. Most patients completed the chiropractic treatment within 6 months [after 3 months: 43% of the patients were still in chiropractic treatment (*N* = 19, 23 missing values); after 6 months: 21% (*N* = 10, 20 missing values); after 12 months: 17% (*N* = 9, 15 missing values)]. With regard to previous treatments in the last year, 4 patients had received infiltrations, 38 patients had undergone physiotherapy (3 of them in combination with training therapy), 4 had received massage therapy, 2 complementary medical treatments, and 6 patients had received multi-disciplinary therapy (physiotherapy and infiltration: *N* = 5, massage and infiltration: N = 1). Of 13 patients, it was unknown whether they had received other treatments. With regard to other therapies in parallel to the chiropractic treatment, 13 patients reported to receive other therapies at the time of chiropractic treatment (physiotherapy: *N* = 10, massage therapy: *N* = 3), 13 reported to have stopped other therapies, and no information about current therapy status was available of the remaining patients.

**Fig. 1:**
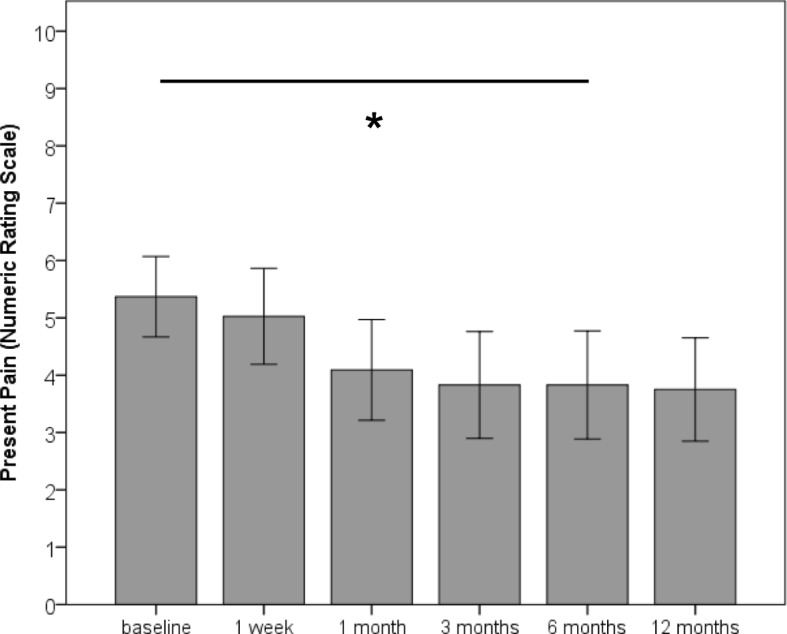
Course of present pain over 6 months after start of chiropractic treatment. Bars represent mean and error bars represent 95% confidence intervals. Present pain was assessed on an 11-point numeric rating scale (NRS). * *p* < 0.05

At baseline, mean NRS for present pain was 5.43 (SD 2.37) and mean BQ was 39.80 (SD 15.16) points (Table 1). NRS for present pain intensity significantly decreased [LMM: F(5, 106.77) = 3.15, *p* = 0.011] to 4.05 (SD 2.88) after 12 months, with significant pairwise-comparison between baseline and 6 months after treatment start (*p* = 0.04) (Fig. 1). This represents a relative reduction of 25.4%. For comparison, the repeated measures ANOVA with imputed values resulted in F-values between F(3.35, 221.24) = 7.76, *p* < 0.001 and F(3.50, 230.68) = 4.77, *p* = 0.002. Post hoc tests showed a significant reduction in present pain intensity 6 months after treatment start. This finding was consistent in all imputations with the exception of one model, which showed significant pain reduction after the first month.

**Table 1:** Course of pain and bio-psycho-social impairment over 12 months after start of chiropractic treatment

	Baseline (SD)	1 week (SD)	1 month (SD)	3 months (SD)	6 months (SD)	12 months (SD)
Present pain (NRS)	5.43 (2.37)	5.14 (2.46)	4.42 (2.81)	4.52 (2.99)	4.09 (2.89)	4.05 (2.87)
BQ total score	39.80 (15.16)	34.91 (14.96)	28.62 (17.96)	27.92 (18.34)	27.96 (18.28)	29.00 (17.90)
BQ 1: average pain over last week	6.30 (2.06)	5.70 (2.07)	4.83 (2.51)	4.80 (2.93)	4.51 (2.75)	4.48 (2.70)
BQ 2: interference with daily activities	5.71 (2.58)	4.97 (2.53)	3.82 (2.90)	3.85 (2.91)	4.07 (3.00)	4.21 (3.02)
BQ 3: interference with recreational, social, and family activities	5.31 (3.24)	4.64 (3.03)	4.48 (3.16)	4.18 (3.19)	3.97 (3.22)	4.14 (3.20)
BQ 4: anxiety	5.60 (3.08)	4.81 (2.61)	3.80 (2.99)	4.02 (2.78)	4.32 (3.07)	4.60 (2.90)
BQ 5: depression	5.08 (3.59)	4.10 (3.08)	3.60 (3.18)	3.34 (3.11)	3.33 (2.96)	3.90 (3.02)
BQ 6: fear avoidance beliefs	6.08 (2.88)	4.99 (3.10)	4.31 (3.16)	4.27 (3.11)	4.35 (3.27)	4.28 (3.32)
BQ 7: locus of control	5.75 (2.70)	5.16 (2.96)	4.10 (2.99)	3.39 (2.68)	3.55 (2.76)	3.93 (2.87)

*BL* Baseline, *BQ* Bournemouth Questionnaire, *NRS* Numeric rating scale

**Fig. 2:**
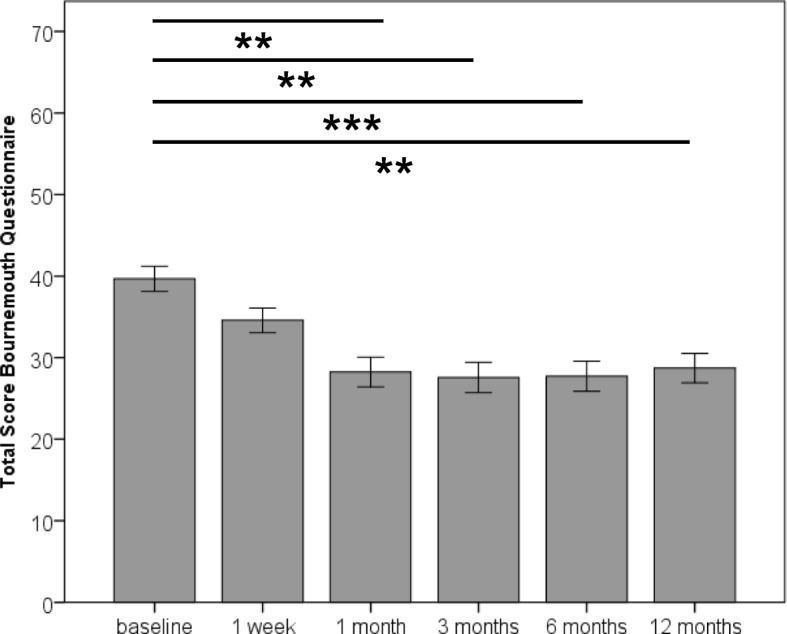
Course of bio-psycho-social impairment (Bournemouth Questionnaire) over 6 months after start of chiropractic treatment. Bars represent mean and error bars represent 95% confidence intervals. ** 0.001 < *p* < 0.01. *** *p* ≤ 0.001

The total score of the BQ significantly diminished [LMM: F(5, 106.47) = 6.55, *p* < 0.001] to 29.00 (SD 17.96) after 12 months, with significant reduction after 1 month (*p* < 0.01) (Fig. 2), corresponding to a relative reduction of 27.2%. Repeated measures ANOVA (with imputed values) resulted in F-values between F(3.43, 226.24) = 14.89, *p* < 0.001 and F(3.49, 230.53) = 17.57, *p* < 0.001. Consistent in all imputations, significant reduction in the BQ total score was observed 1 month after start of the chiropractic treatment, while four of five models showed significant reduction in BQ total score after 1 week.

All single BQ items except the item ‘interference of back pain with ability to take part in recreational, social and family activities’ [LMM: F(5,101.64) = 1.55, *p* = 0.182; imputations: F-values between F(4.00, 263.77) = 3.65, *p* = 0.007 to F(3.80, 250.87) = 4.54, *p* = 0.002; relative reduction = 22.0%] significantly decreased over time: ‘average pain over the last week’ [LMM: F(5,113.78) = 7.46, *p* < 0.001; imputations: F-values between F(3.35, 220.79) = 12.49, *p* < 0.001 to F(3.79, 250.14) = 15.25, *p* < 0.001; relative reduction = 28.9%], ‘interference of pain with daily activities over the last week’ [LMM: F(5, 106.05) = 5.68, *p* < 0.001; imputations: F-values between F(4.14, 273.38) = 10.09, *p* < 0.001 to F(4.23, 279.00) = 11.41, p < 0.001; relative reduction = 26.3%], ‘feeling anxious’ [LMM: F(5, 108.23) = 3.23, *p* = 0.009; imputations: F-values between F(3.94, 260.13) = 5.94, *p* < 0.001 to F(3.80, 250.57) = 7.34, p < 0.001; relative reduction = 18.4%], ‘feeling depressed’ [LMM: F(5, 106.08) = 2.96, *p* = 0.015; F-values between F(3.56, 235.08) = 6.41, *p* < 0.001 to F(3.77, 248.71) = 7.44, *p* < 0.001; relative reduction = 23.2%], ‘impact of work on back pain’ [LMM: F(5, 97.41) = 4.38, *p* = 0.001; imputations: F-values between F(3.90, 257.53) = 6.13, *p* < 0.001 to F(3.77, 248.52) = 7.09, p < 0.001; reduction = 29.6%] and ‘own ability to control back pain’ [LMM: F(5, 111.20) = 7.90, *p* < 0.001; imputations: F-values between F(3.94, 259.77) = 11.70, p < 0.001 to F(4.19, 276.33) = 13.60, *p* < 0.001; relative reduction = 31.7%]. LMM revealed significant reduction in all items within the first month of treatment (‘average pain over last week’: *p* < 0.01; ‘interference with daily activities’, ‘anxiety’, ‘fear avoidance beliefs’, ‘locus of control’: *p* < 0.05) apart from ‘depression’ that did not decrease before 3 months after treatment start (Table 1). Using imputations, all items (items ‘average pain over last week’, ‘interference with daily activities’, ‘fear avoidance beliefs’, ‘locus of control’: *p* < 0.01; items ‘anxiety’ and ‘depression’: *p* < 0.05) significantly decreased within the first month after treatment start with the exception ‘interference with recreational, social, and family activities’ that did not decrease before 6 months (*p* < 0.05).

The proportion of patients reporting overall improvement significantly increased from 23% (13/56 patients, 11 missing values) after 1 week to 47% (27/57, 10 missing values) after 1 month, when it stabilized (*p* = 0.004): 56% of the patients (33/59, 8 missing values) reported overall improvement after 3 and 6 months, and 44% (24/54; 13 missing values) after 12 months. Overall improvement was not associated with reduction in pain (NRS), but with bio-psycho-social impairment (BQ) from 1 month onward after start of chiropractic treatment (Table 2).

**Table 2:** Model 1: Prediction of self-reported overall improvement by changes in present pain (NRS) and Bournemouth Questionnaire (BQ)

	B (SE)	Exp B (Odds Ratio)	95% CI Exp B	*p*
PGIC 1 week: Nagelkerke R^2^ = 0.03–0.10				
Change NRS present pain	−0.03 (0.15)	0.97	0.72–1.31	0.842
Change BQ total score	0.04 (0.03)	1.04	0.98–1.11	0.178
PGIC 1 month: Nagelkerke R^2^ = 0.23–0.30				
Change NRS present pain	0.16 (0.11)	1.17	0.95–1.44	0.134
Change BQ total score	0.06 (0.03)	1.06	1.00–1.12	*0.039*
PGIC 3 months: Nagelkerke R^2^ = 0.31–0.48				
Change NRS present pain	0.04 (0.14)	1.05	0.80–1.37	0.743
Change BQ total score	0.10 (0.04)	1.11	1.02–1.20	*0.013*
PGIC 6 months: Nagelkerke R^2^ = 0.42–0.46				
Change NRS present pain	0.24 (0.12)	1.27	0.99–1.61	0.056
Change BQ total score	0.08 (0.03)	1.08	1.02–1.15	*0.016*
PGIC 12 months: Nagelkerke R^2^ = 0.32–0.43				
Change NRS present pain	0.04 (0.14)	1.04	0.79–1.37	0.794
Change BQ total score	0.09 (0.04)	1.10	1.02–1.18	*0.021*

Logistic regressions with PGIC (0 = not improved, 1 = improved) of each time point as dependent variable and the changes in NRS and BQ as independent variables

*BQ* Bournemouth Questionnaire, *NRS* Numeric rating scale, *PGIC* Patient Global Impression of Change

Model 2 (not run for overall improvement after 1 week because pain intensity and bio-psycho-functional impairment emerged as not significant from model 1), explained 20–39% (Nagelkerke R^2^ = 0.20–0.39) of variance after 1 month, 46–62% after 3 months, 50–59% after 6 months and 41–54% after 12 months. The only significant single item was the item ‘fear avoidance beliefs’ that was significant after 6 months (*p* = 0.023).

## Discussion

In this population of patients referred from the spine division to the chiropractic teaching clinic, NRS for present pain and BQ total score for bio-psycho-social impairment diminished by about 25% within the first 12 months after the start of chiropractic treatment. The BQ total score showed significant reduction within 1 month, while present pain intensity did not decrease before 6 months after treatment start. Most BQ items significantly improved within the first month, apart from the items ‘ability to take part in recreational, social and family activities’ that did not improve at all and the item ‘depression’ that did not improve before 3 months. Improvement of the single BQ items was between 18% (‘anxiety’) and 32% (‘own ability to control back pain’). The observed overall improvement (PGIC ‘better’ or ‘much better‘) from 1 month onwards, reported by approximately half of the patients, was associated with a significant reduction in bio-psycho-social impairment (BQ total score) at each point in time, but not with pain reduction (NRS), and could, apart from the item ‘fear avoidance beliefs’ at 6 months, not be related to single BQ-items.

For the patients in this study, the reduction in pain intensity seemed of minor importance for their rating of overall improvement, which is in accordance with previous literature [22]. In contrast, the total reduction in bio-psycho-social impairment seemed to account for the self-perceived overall improvement in this population of chronic pain patients. Interestingly, this was not attributable to a single BQ-item. These findings underline the importance of complementing pain assessment by a comprehensive assessment of bio-psycho-social impairment, such as the BQ, to describe progress in the treatment of such chronic pain patients.

In contrast to acute patients, where present pain intensity substantially diminishes within the first week of treatment [23], present pain (NRS) did not significantly improve before 6 months after treatment start. Interestingly however, although it was not associated with overall improvement, average pain over the last week (the first BQ item) significantly improved within the first month of treatment. This discrepancy raises the issue of which recall period is best to be used for assessing pain. In the literature, different recall periods are used (e.g. present pain [24], pain in the past 24 h [25], pain in the past 4 weeks [24], or a combined measure [24]), and often the recall period is not specified [26, 27]). A qualitative study on the patient perspective on measures for chronic pain identified difficulty averaging pain as one of the four main themes [28]. The discrepancy between present and average pain observed in the present study underlines the importance of being precise when assessing pain.

Average pain over the last week and present pain decreased by 29 and 25%, respectively within the first 12 months after the start of chiropractic treatment. This is close to 30%, which is considered a clinically meaningful change for chronic musculoskeletal pain [25, 27, 29]. Thus even after 12 months, when chiropractic treatment had been terminated for about 80% of the patients, pain intensity of this chronic patient population stayed significantly reduced on average. These findings are in line with previous literature reporting pain relief in the first 3 months after start of chiropractic treatment, which remained stable up to 12 months for acute and chronic LBP patients [30]. For the BQ total score, the minimal clinically important change (MCIC) for chronic LBP patients has been reported to be 18 points [31]. In contrast, patients in the present study reported less reduction in BQ, although approximately half of them reported overall improvement. The main reason for this divergence might be the different setting: patients recruited in a chiropractic practice [31] versus patients referred from orthopedic surgeons, the latter resulting in a patient sample of reduced general health with higher bio-psycho-social impairment (BQ score of 40 compared to 34 [31]).

The proportion of patients who indicated overall improvement was slightly lower than in a similar study that recruited chronic LBP patients from multiple chiropractic practices throughout Switzerland, which reported 69% improved patients 3 months after start of chiropractic treatment [17]. Again, this difference might be explained by the different patient sample in the present study: patients were in a worse health state as mirrored in poorer general health (52% vs. 60% good health), more patients with LBP lasting for longer than 1 year (70% vs. 45%), and in a higher proportion of patients taking pain medication at baseline (35% vs. 28%) compared to the other study [17]. Altogether, these findings support the hypothesis that the patients of the present study were not average, but severe chronic LBP patients. In addition, this study focused on patients treated in a chiropractic teaching clinic by chiropractic students with limited clinical experience. Despite these two challenges, the proportion of clinically relevantly improved patients after eight chiropractic sessions (median) was high.

Nevertheless, it is important to note that these results do not inform about the efficacy of chiropractic treatment for several reasons: Many patients had other therapies besides chiropractic treatment, foremost physiotherapy. Furthermore, this study did not include a natural history control group and did not track patients who returned to orthopedic care. Another limitation is that the study reports associations between outcome measures and no conclusions about causation can be drawn.

## Conclusions

These results illustrate that chronic LBP patients with long-lasting pain, reduced general health and high bio-psycho-social impairment, referred from spine surgery to a chiropractic teaching clinic, benefit from being co-managed by surgeons and chiropractors. These findings emphasize the advantages and importance of close collaboration between the two disciplines in order to provide optimal care for chronic back pain patients. The consequence might be an even closer collaboration between the disciplines with focus on spine medicine. In Switzerland, this has now been established at the joint University Spine Center Zurich, where multiple disciplines (Orthopedic and Neuro-Surgery, Neurology, Neuro-Urology, Chiropractic medicine, Rheumatology, Anesthesiology and Radiology) are closely working together for optimal patient care.

## Abbreviations

BQ: Bournemouth Questionnaire
LBP: Low back pain
NRS: Numeric Rating Scale
PGIC: Patient’s Global Impression of Change
